# Structure and Biophysical Properties of a Triple-Stranded Beta-Helix Comprising the Central Spike of Bacteriophage T4

**DOI:** 10.3390/v7082839

**Published:** 2015-08-18

**Authors:** Sergey A. Buth, Laure Menin, Mikhail M. Shneider, Jürgen Engel, Sergei P. Boudko, Petr G. Leiman

**Affiliations:** 1 Institute of Physics of Biological Systems, École Polytechnique Fédérale de Lausanne (EPFL), BSP 415, 1015 Lausanne, Switzerland; E-Mails: sergii.buth@epfl.ch (S.A.B.); mm_shn@mail.ru (M.M.S.); 2 Service de Spectrométrie de Masse, ISIC, EPFL, BCH 1520, 1015 Lausanne, Switzerland; E-Mail: laure.menin@epfl.ch; 3 Shemyakin-Ovchinnikov Institute of Bioorganic Chemistry, Laboratory of Molecular Bioengineering, 16/10 Miklukho-Maklaya St., 117997 Moscow, Russia; 4 Department of Biophysical Chemistry, Biozentrum, University of Basel, Klingelbergstrasse 70, CH-4056 Basel, Switzerland; E-Mail: juergen.engel@unibas.ch; 5 Department of Biological Sciences, Purdue University, 915 W. State Street, West Lafayette, IN 47907-2054, USA; 6 The Research Department, Shriner’s Hospital for Children, 3101 Sam Jackson Park Road, Portland, OR 97239, USA; 7 Department of Biochemistry and Molecular Biology, Oregon Health and Science University, 3181 Sam Jackson Park Road, Portland, OR 97239, USA

**Keywords:** β-helical proteins, fibrous proteins, protein folding, X-ray crystallography, fatty acid, mass spectrometry, intrinsic protein fluorescence, protein stability, amyloid-like structure, low complexity amino acid sequence

## Abstract

Gene product 5 (gp5) of bacteriophage T4 is a spike-shaped protein that functions to disrupt the membrane of the target cell during phage infection. Its C-terminal domain is a long and slender β-helix that is formed by three polypeptide chains wrapped around a common symmetry axis akin to three interdigitated corkscrews. The folding and biophysical properties of such triple-stranded β-helices, which are topologically related to amyloid fibers, represent an unsolved biophysical problem. Here, we report structural and biophysical characterization of T4 gp5 β-helix and its truncated mutants of different lengths. A soluble fragment that forms a dimer of trimers and that could comprise a minimal self-folding unit has been identified. Surprisingly, the hydrophobic core of the β-helix is small. It is located near the C-terminal end of the β-helix and contains a centrally positioned and hydrated magnesium ion. A large part of the β-helix interior comprises a large elongated cavity that binds palmitic, stearic, and oleic acids in an extended conformation suggesting that these molecules might participate in the folding of the complete β-helix.

## 1. Introduction

Bacteriophage T4 uses a large multicomponent organelle, called a tail, for recognition and attachment to an *Escherichia coli* cell [1,2]. Gene product (gp) 5 is critical for the assembly of the tail’s baseplate [3,4,5] and for the tail function during infection [6]. A spike-shaped trimeric protein, gp5 forms the centerpiece of the baseplate [7]. It has long been hypothesized to function as a piercing needle with which the phage disrupts the multilayered host cell envelope upon attachment to the cell surface and subsequent sheath contraction [7]. The amino acid sequence of gp5 contains 575 residues that form three domains connected by long linkers. Residues 1–129, 174–339, and 389–575 comprise the N-terminal OB-fold domain, the middle lysozyme domain, and the C-terminal β-helical domain, respectively [7]. The latter is a 100 Å-long and 30 Å-wide triple-stranded β-helix formed by three fully interdigitated polypeptide chains.

Many virus and phage fibrous proteins contain triple-stranded β-helical regions or domains [8,9,10,11,12,13,14,15,16,17,18,19,20], but gp5 β-helix is by far the longest and most regular of all such structures. Besides, two properties of gp5 β-helix set it apart from other interdigitated oligomeric β-helices: it shows a well-defined repeating motif VxGxxxxx [7,21], and it can fold on its own [5]. These features make gp5 β-helix a valuable protein engineering tool that can serve as a trimerization domain or as a building unit in supramolecular complexes. Notably, the repeating motif allows for manipulating the length of the helix by extending it with a pseudo-repetitive sequence of interest [22].

Several studies describing the use of gp5 β-helix in the assembly of large oligomeric structures have been reported [23,24,25,26]. However, many structural details of gp5 β-helix and its folding properties have been either uncharacterized or characterized incorrectly. These properties cannot be derived from other systems because folding and structure of oligomeric fibrous proteins are extremely poorly understood and no protein with a topology similar to that of gp5 β-helix has ever been characterized in sufficient detail.

To address these issues and to expand the scope of possible applications, we analyzed the structure and biophysical properties of gp5 β-helix and its deletion mutants. We found that the central part of this intertwined protein, but now one of its extremities as is the case with other fibrous proteins, is soluble suggesting that it can fold on its own in the cell and, possibly, could initiate folding of the full-length protein. Interestingly, instead of possessing a tightly packed core, the interior of this region of the β-helix comprises a large cavity that binds fatty acids in the full-length structure. Gp5 β-helix demonstrated an exceptional resistance to denaturation by heat and chaotropic agents and, for example, maintained its structure in 6 M guanidinium at 50 °C. These findings establish a framework for using gp5-like β-helices in protein engineering and give experimental information about folding and detailed composition of an oligomeric protein with a complex intertwined topology. 

## 2. Materials and Methods

### 2.1. Cloning, Expression and Purification

#### 2.1.1. Gp5β-ABC, Gp5β-BC, Gp5β-C, and Gp5β-B Fragments

**Figure 1:**
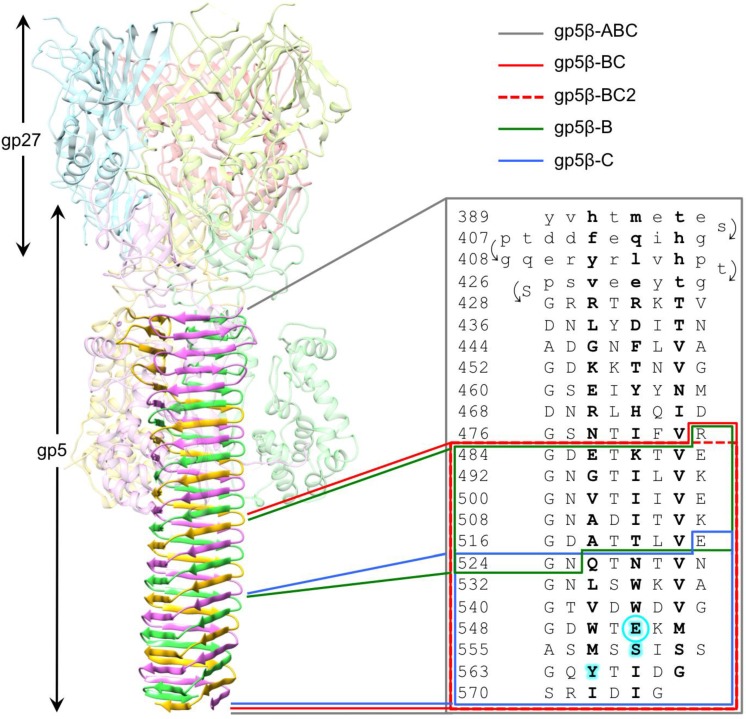
Design of gp5 β-helix fragments. Ribbon diagram of the gp5-gp27 complex shows the location of the gp5 β-helix within the complex. Residues comprising the N-terminal part of the β-helical domain with an antiparallel β-sheet topology are shown in small letters. The corkscrew part of the β-helix is in capital letters. Residues pointing inwards are highlighted in bold. Residues involved in binding of the buried metal ion and associated water molecules are highlighted with a cyan background with the critical glutamates (Glu552) emphasized with an additional circle.

Fragments of T4 gene 5 corresponding to gp5β-ABC, gp5β-BC, gp5β-C, and gp5β-B (Figure 1) were PCR amplified and cloned into the pHisTrx2 [27] vector using restriction sites *BamHI* and *EcoRI*. The vector is designed to express fusion constructs containing an N-terminal His-tag, thioredoxin A, linker with thrombin cleavage site and a fragment of interest. The DNA inserts were verified by Sanger dideoxy DNA sequencing.

The recombinant proteins were expressed as fusion constructs at 37 °C in the *E. coli* BL21 (DE3) host strain (Novagen, Darmstadt, Germany) after IPTG induction to a final concentration of 1 mM. The SeMet mutant of R483 was expressed in modified M9 medium in the presence of Se-methionine using the B834 (DE3) strain of *E. coli*. Purification of the fusion proteins by immobilized metal affinity chromatography on a HisTrap^TM^ HP column (Amersham Biosciences, Little Chalfont, UK) and separation of gp5 mutants after thrombin cleavage were carried out according to the manufacturer’s instructions. Before thrombin cleavage, the fusion proteins were additionally purified on an anion exchange HiTrap^TM^ Q HP column (Amersham Biosciences) or monoQ column (GE Healthcare Life Sciences, Little Chalfont, UK). The cleavage was performed at 20 °C for 16 h with thrombin (Novagen).

#### 2.1.2. Gp5β-BC2 Fragment

The fragment of gene 5 corresponding to gp5β-BC2 was cloned *in cis* and upstream of full length gene 5.4 into the pEEva2 plasmid (a derivative of pET23a). Gp5β-BC2 carried an N-terminal His-tag separated from the rest of the sequence by a TEV-cleavage site (ENLYFQG) and a linker (SGS). Gp5.4 was tagless. The plasmid was transformed into the B834 (DE3) strain of *E. coli*. The transformed cells were grown at 37 °C in the LB medium, complemented with ampicillin at the concentration of 200 μg/mL until the optical density reached the value of 0.6–0.8 at 600 nm. The culture was cooled on ice to the temperature of 18–20 °C and protein expression was induced by addition of IPTG to a final concentration of 1 mM. After overnight incubation at 18 °C (approximately 16 h), the cells were harvested and lysed by sonication. Cleared lysate was loaded onto the 5 mL GE HisTrap FF Crude column (GE Healthcare Life Sciences). Protein was eluted using two-step gradients on an AKTApurifier 100 system (GE Healthcare Life Sciences). The fractions of the elution peak were pulled together and dialyzed overnight with simultaneous TEV His-tag cleavage. Digested protein was further purified with ion-exchange chromatography (GE Mono Q 10/100 GL column connected to an AKTApurifier 100 system). Selected fractions of the ion-exchange chromatography were analyzed on SDS-PAGE gel. The protein of interest was further purified by size exclusion chromatography using a GE HiLoad 16/60 Superdex 200 PG (GE Healthcare Life Sciences) column connected to the AKTApurifier 100 system (GE Healthcare Life Sciences).

### 2.2. Circular Dichroism and Fluorescence Analysis

The circular dichroism spectra were acquired on a Cary 61 spectropolarimeter (Varian, Monrovia, CA, USA) equipped with a temperature-controlled 1-mm path length quartz cell. The spectra were normalized for concentration and path length to obtain the mean molar residue ellipticity after subtraction of the buffer contribution. All spectra were recorded in either a 10 mM TrisHCl buffer, pH 8.0 or a 20 mM sodium phosphate buffer, pH 6.0 or 8.0 and no salt the thermal unfolding was monitored by the change in the mean molar residue ellipticity at a fixed wavelength of 215 nm.

Fluorescence spectra were recorded on a LM8000C instrument (SLM Instruments, Rochester, NY, USA) with modified electronics (ISS Corp., Chicago, IL, USA) using 1 cm × 1 cm quartz cells (Hellma, Müllheim, Germany). Protein samples were in 50 mM TrisHCl, pH 8. Emission spectra were obtained by excitation of protein samples at 280 nm at 30 °C.

### 2.3. Analytical Ultracentrifugation

Sedimentation equilibrium experiments were performed on a Beckman Optima XL-A analytical ultracentrifuge (Beckman Instruments, Fullerton, CA, USA) equipped with 12-mm Epon double-sector cells in an An-60 Ti rotor. The proteins were analyzed in 10 mM sodium phosphate buffer (pH 7.4) containing 150 mM NaCl at +6 °C. Protein concentrations were adjusted to 0.12–0.25 mg/mL. Sedimentation equilibrium scans were carried out at 28,000 rpm. Molecular masses were evaluated using log *A* *vs.* r^2^ plots, where *A* is the absorbance and r is the distance from the rotor center [28]. A partial specific volume of 0.73 mL/g was used for all calculations.

### 2.4. Crystallization and Structure Determination

#### 2.4.1. Gp5β-BC

For crystallization, gp5β-BC was brought to a concentration of 20 mg/mL in 10 mM Tris-HCl pH 8.0 and mixed with a crystallization solution containing 11% MPD, 50 mM CaCl_2_, and 100 mM Na-Acetate pH 5.2. The crystals were grown using the hanging drop method (2 μL drop size) in 24 deep-well plates. Crystals of 0.3 mm × 0.2 mm × 0.5 mm appeared after 4 days of incubation at +18 °C. For data collection, the crystals were quickly dipped into the cryo-protector solution (25% MPD, 50 mM CaCl_2_, and 100 mM Na-Acetate, pH 5.2) and transferred to a vaporized liquid nitrogen stream at 100 K. Indexing, integrating and scaling were done using HKL2000 [29]. The structure was solved by molecular replacement using the MOLREP program [30] from CCP4 program suite [31] and a fragment comprising residues 483–575 of the atomic model of gp5 from the gp5-gp27 structure [7]. The structure was refined with SHELXL [32] and Coot [33]. The details of data reduction and refinement are given in Table 1. The structure of gp5β-BC fragment was deposited into the Protein Data Bank under the accession number 4JJ2.

**Table 1:** Crystallographic statistics.

	Gp5β-BC	Gp5β-BC2
Data Collection		
Beamline	PXIII (SLS)	PXI (SLS)
Detector	MARMOSAIC 225mm CCD	DECTRIS PILATUS 6M
Wavelength (Å)	0.900	1.000
Oscillation angle (°)	1.0	0.25
Number of frames	360 + 360 *	990
Space group	C222_1_	P2_1_
Cell dimensions: *a*, *b*, *c* (Å), α, β, γ (°)	57.61, 72.76, 130.49, 90.00, 90.00, 90.00	110.29, 73.79, 111.60, 90.00, 113.39, 90.00
Number of polypeptide chains in the asymmetric unit	3	9
Resolution interval (Å)	50.0–1.3	50.0–2.0
R_sym_ (%)	0.069 (0.299)^#^	0.095 (0.363)
<I/σ_I_>	11.3 (3.6)	6.7 (2.4)
Completeness (%)	99.59 (87.0)	96.6 (95.9)
Redundancy	10.2 (4.3)	2.4 (2.5)
**Refinement**		
Number of reflections		
Working	69991	110537
Test	4689	5818
R_work_/R_free_	0.153/0.178	0.219/0.279
B-factor (Å^2^)	19.4	35.3
R.m.s. deviations		
Bond lengths (Å)	0.0169	0.019
Bond angles (°)	0.0413	1.849
Number of atoms		
Protein	2259	12217
Solvent and ligands	388	992
Ramachandran plot (%)		
Most favored	97.4	98.9
Additionally allowed	2.6	1.1
Outliers	0.0	0.0
**PDB ID**	**4JJ2**	**4OSD**

* Two acquisitions for high and low resolution were performed on the same crystal; ^#^ Data in parenthesis represent statistics for the highest resolution shell.

#### 2.4.2. Gp5β-BC2

Crystals of gp5β-BC2 were obtained by mixing 1.25 μL of protein at a concentration of 22 mg/mL in 10 mM Tris-HCl pH 8.0, 150 mM NaCl with 1.25 μL of reservoir solution and allowed to equilibrate against 500 μL of 22% PEG 4000, 200 mM Li_2_SO_4_, 100 mM Tris-HCl pH 8.5 at 18 °C. Prism-like crystals appeared in about 5 days and continued to grow for another week reaching dimensions of 0.2 mm × 0.15 mm × 0.1 mm. The mother liquor served as a cryo-protector. The diffraction data was indexed, integrated and scaled with XDS [34,35]. The details are summarized in Table 1. The structure of the protein was determined by molecular replacement using the program PHASER [36] and the refined structure of gp5β-BC as a search model. The model was refined by interactive cycles of building with Coot [33] and refinement with REFMAC5 [37] using NCS. The structure of gp5β-BC2 fragment was deposited into the Protein Data Bank under the accession number 4OSD.

### 2.5. Extraction of Internal Compounds from gp5β-BC

To extract the inclusion compounds from the gp5β-BC trimer a modified Folsch procedure was applied [38]. Aqueous solution of gp5β-BC crystals (approximate volume 300 μL) was mixed in a 1.5 mL low-binding reaction tube (Eppendorf, Hamburg, Germany) with 750 μL of the extraction mixture composed of chloroform/methanol, 2:1 *v/v*. The tube was mixed with a vortex mixer at 16,000 rpm for about 3 min. Then organic phase was transferred to another tube and dried under vacuum down to a volume of 150 μL. This sample was then used for GC-MS analyses.

### 2.6. Mass Spectrometry Analyses

#### 2.6.1. ESI-QTOF-MS

In order to determine the molecular weight of the inclusion compounds, several gp5β-BC samples were subjected to Electrospray-Ionization mass-spectrometry. ESI-MS data were acquired on a Q-Tof Ultima mass spectrometer (Waters, Milford, MA, USA) operated in the positive ionization mode and fitted with a standard Z-spray ion source equipped with the Lock-Spray interface. The experimental parameters were set as follows: capillary voltage, 3.5 kV; sample cone, 50 V; source temperature, 80 °C; desolvation temperature, 200 °C; acquisition window, *m*/*z* 500–2500 in 1 s. The external calibration was carried out with a solution of phosphoric acid at 0.01% introduced through an orthogonal ES probe. Data were processed using the MassLynx 4.1 software. The protein was diluted in H_2_O.

ESI-MS analysis of gp5β-BC using its crystallization-grade stock solution at a concentration of 20 mg/mL in water did not reveal any dominant peaks in the low molecular weight range of the spectrum. To reduce the amount of low molecular weight impurities, gp5β-BC was purified by crystallization: crystals of gp5β-BC were collected, washed in the crystallization solution free from protein, and dissolved in distilled water. Then, the crystals were dialyzed against distilled water by 3 repeated buffer exchanges using a Millipore microconcentration device (Merck Millipore, Darmstadt, Germany) with a cutoff of 10 kDa. The final concentration of thus purified protein was about 5 mg/mL. This sample was then diluted by H_2_O (native conditions) or by a CH_3_CN/H_2_O/HCOOH mixture with a ratio of 50:49.9:0.1 (denaturing conditions). In order to extract the internal compounds, these specimens were subjected to in-source denaturation by increasing the sample cone voltage or sample chamber temperature.

A negative control MS experiment was performed on a crystallization solution without protein to obtain the characteristic MS fingerprint of the mother liquor for subtraction from protein samples datasets.

#### 2.6.2. GC-IE-MS

GC-MS analyses were carried out on a Varian 1200 L quadrupole MS/MS analyzer coupled to a CP-3800 gas chromatograph. A capillary column FactorFour^TM^ VF-5ms (5% phenyl-methyl 95% dimethylpolysiloxane column, 0.25 mm × 30 m) was used with helium as carrier gas. One microliter of sample was injected at an oven temperature of 50 °C, then the oven temperature was increased to 150 °C at 5 °C/min, then subsequently to 300 °C at 20 °C/min. Ionization used was Electron Impact (EI) using an electron energy of 70 eV, over a mass range of 50–500 *m*/*z* with a dwell time of 0.5 scan/s.

### 2.7. Sequence Analysis

Gp5 from the following bacteriophages were used in the creation of Figure 13: T4 (NP_049757.1), *Escherichia* phage ECML-134 (YP_009102621.1), *Yersinia* phage PST (AGR46096.1), *Escherichia* phage wV7 (YP_007004892.1), *Enterobacteria* phage RB3 (YP_009098534.1), *Enterobacteria* phage RB51 (YP_002854107.1), *Enterobacteria* phage AR1 (BAI83163.1), *Enterobacteria* phage IME09 (YP_007004528.1), *Shigella* phage Shfl2 (YP_004415044.1), *Enterobacteria* phage RB32 (YP_803092.1), *Enterobacteria* phage RB14 (YP_002854485.1), *Enterobacteria* phage vB_EcoM_ACG-C40 (YP_006986702.1), *Yersinia* phage phiD1 (CCI89046.1), *Enterobacteria* phage vB_EcoM_VR20 (AIZ02221.1), *Enterobacteria* phage vB_EcoM-VR7 (YP_004063846.1), *Enterobacteria* phage vB_EcoM_VR26 (AIZ02797.1), *Enterobacteria* phage vB_EcoM_VR25 (AIZ02510.1), *Enterobacteria* phage vB_EcoM_VR5 (AIZ01937.1), *Enterobacteria* phage JS10 (YP_002922494.1), *Enterobacteria* phage IME08 (YP_003734291.1), *Enterobacteria* phage JS98 (YP_001595278.1), *Salmonella* phage S16 (YP_007501180.1), *Salmonella* phage STML-198 (AFU64090.1), *Escherichia* phage vB_EcoM_JS09 (YP_009037344.1), *Shigella* phage Shf125875 (YP_009100697.1), *Escherichia* phage vB_EcoM_PhAPEC2 (YP_009056740.1), *Enterobacter* phage PG7 (YP_009005432.1), *Enterobacteria* phage CC31 (YP_004010012.1), *Enterobacteria* phage RB69 (NP_861854.1), *Yersinia* phage phiR1-RT (YP_007235983.1), *Acinetobacter* phage 133 (YP_004300735.1), *Acinetobacter* phage Ac42 (YP_004009522.1), *Shigella* phage SP18 (YP_003934787.1), *Enterobacteria* phage Bp7 (YP_007004296.1), *Acinetobacter* phage Acj9 (YP_004010297.1), *Acinetobacter* phage ZZ1 (YP_006489164.1), *Acinetobacter* phage Acj61 (YP_004009776.1).

## 3. Results

### 3.1. Design of β-Helix Fragments

The 2.9 Å resolution crystal structure of the complete gp5 trimer [7] (Figure 1) showed that the first four strands of the β-helix form an antiparallel β-sheet (residues 389–427), which is then extended by a corkscrew-like intertwined part (residues 428–575), and that the N-terminal half of the β-helix (residues 389–482) interacts with the lysozyme domain of gp5 and thus is partially shielded from solvent. The interior of the β-helix was found to be sandwich-like with hydrophobic, polar, and charged residues either completely filling the volume or forming large cavities. Surprisingly, the β-helix did not have a well-defined buried hydrophobic core, except for the C-terminal tip, although this part also contained a hydrophilic cavity, raising a question of which region of this complex structure plays the most important role in folding and trimerization. We decided to investigate this problem by creating several staggered and overlapping deletion mutants taking the interior architecture of the β-helix into consideration. The following fragments were created: gp5β-ABC–the entire β-helical domain (residues 389–575), gp5β-BC—its solvent-exposed part (residues 483–575), gp5β-C—its C-terminal tip (residues 523–575), gp5β-B—the middle region of the solvent-exposed part that comprised about 1/3rd of its length (residues 483–525 with N525 mutated to Y) (Figure 1). Notably, the sequences of the gp5β-B and gp5β-C mutants added together comprised the sequence of the gp5β-BC fragment. In a later study, the gp5β-BC mutant was shortened by one amino acid (R483 was removed) to give rise to a fragment containing residues 484–575 (gp5β-BC2). It was expressed using a different expression vector requiring changes in the purification procedure. This mutant was used as a platform for creating chimeras of gp5 for purification of PAAR repeat proteins in a separate study [22].

### 3.2. Secondary Structure and Oligomeric State of β-Helix Fragments

**Figure 2:**
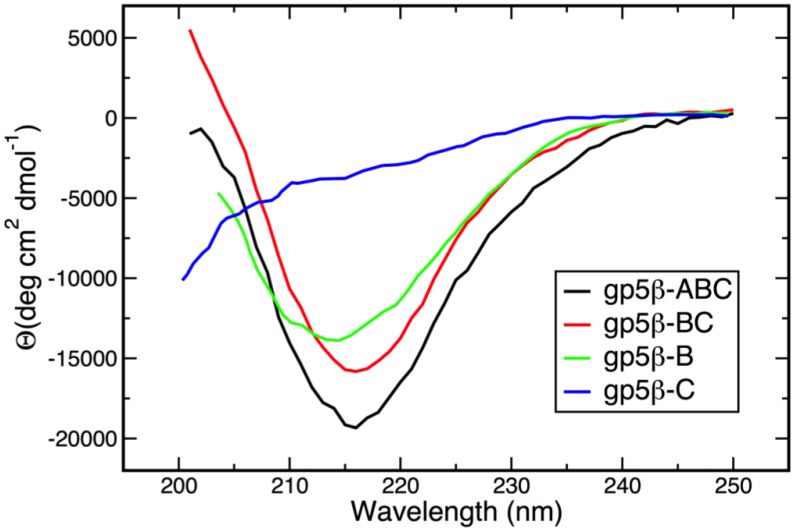
Circular dichroism spectra of gp5 β-helix fragments used in this study. CD spectra were recorded in 10 mM TrisHCl buffer, pH 8.0 at 25 °C.

Circular dichroism (CD) spectra of gp5β-ABC, gp5β-BC, gp5β-C, and gp5β-B are presented in Figure 2. The gp5β-BC2 fragment was not studied because it is virtually identical to gp5β-BC. All mutants except gp5β-C have a pronounced minimum at ~215 nm that is a characteristic feature of β-structure [39]. The CD spectrum of gp5β-C is typical for random coil [39] indicating its inability to fold into a β-helical structure.

The oligomerization state of the two shortest fragments with a well-defined β-structural architecture—gp5β-BC and gp5β-B—was analyzed by analytical ultracentrifugation. The sedimentation equilibrium molecular mass of gp5β-BC was found to be 32 ± 3 kDa, consistent with a trimeric structure (the calculated molecular mass of the monomer is 9.9 kDa). The mass of gp5β-B was found to be 24 ± 3 kDa, which is over five times the weight of the monomer (the calculated monomer’s mass is 4.7 kDa), suggesting that this mutant might form a hexamer, most likely a dimer of trimers. The molecular mass of the gp5β-BC fragment determined by size exclusion chromatography was 28.7 ± 0.2 kDa. Notably, in the crystalline state, two gp5β-BC trimers form a dimer via end-on association of their C-terminal hydrophobic tips (see below). It is possible that the gp5β-B mutant forms a similar structure in solution.

### 3.3. Thermal and Chemical Stability of gp5β-BC

Gp5β-BC displays some rather unusual thermal and chemical denaturation properties, which were analyzed by monitoring its distinctive β-structural CD spectrum and intrinsic fluorescence at different temperatures and in the presence of a denaturing agent (guanidinium).

**Figure 3:**
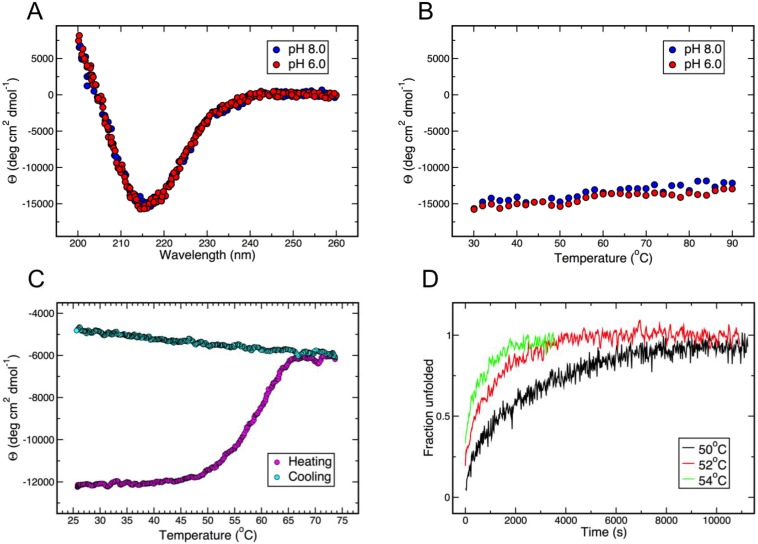
The gp5β-BC fragment is extremely resistant to thermal and chemical denaturation. (**A**) CD spectra in 20 mM Sodium Phosphate buffer at pH 6.0 and 8.0; (**B**) Temperature-induced unfolding is monitored by the depth of the β-structure signature peak at 215 nm; (**C**) Thermal denaturation in the presence of 6 M guanidinium is irreversible; (**D**) Kinetics of unfolding in 6 M guanidinium after incubation at different elevated temperatures.

Gp5β-BC is a very stable protein and maintains its β-structure when heated up to 90° in 20 mM sodium phosphate buffer at pH 6.0 and 8.0 (Figure 3A,B). The protein does not denature in 6 M guanidinium when heated up to 50 °C and starts to unfold in the range of 50–65 °C (Figure 3C), where the unfolding rate shows a strong dependence on temperature (Figure 3D). The unfolding is irreversible and cooling does not lead to β-structure recovery (Figure 3C). Complete unfolding can be achieved after prolonged incubation at 50° (Figure 3D). Renaturation of guanidinium- and heat-denatured gp5β-BC is possible only after complete removal of guanidinium. Two sequential dialyses against 20 mM sodium phosphate buffer at pH 8.0 were able to restore the characteristic β-structure CD spectra.

**Figure 4:**
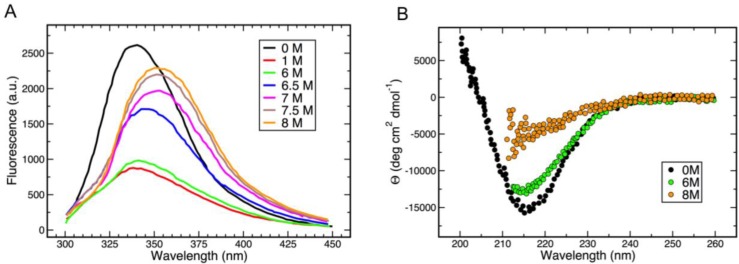
Probing the conformation of gp5β-BC with guanidinium. (**A**) gp5β-BC fluorescence in the presence of an increasing concentration of guanidinium; (**B**) gp5β-BC CD spectra in the presence of 0, 6 and 8 M guanidinium.

Despite preserving its β-structure in the presence of 6 M guanidinium, addition of a relatively small amount of this denaturing agent (starting from 0.1 M) leads to strong quenching of gp5β-BC intrinsic fluorescence (Figure 4A). Nevertheless, consistent with the secondary structure preservation, the fluorescent spectra of gp5β-BC with guanidinium concentrations of up to 6 M are very similar. As the concentration of guanidinium is further increased to 8 M, the gp5β-BC fluorescence rapidly increases almost to the level of the guanidinium-free sample (Figure 4A). Notably, the peaks become broader and shift to longer wavelength values showing that buried hydrophobic residues become solvent exposed. CD confirms that the protein loses most of β-structure (Figure 4B).

### 3.4. Crystal Structure of gp5β-BC and gp5β-BC2

The structure of gp5β-BC is very similar to the corresponding fragment of the full-length protein. However, the much-improved resolution (1.3 Å *vs.* 2.9 Å) allows for precise description of several previously incorrectly identified or uncharacterized features of its interior. PISA analysis [40] shows that each chain of gp5β-BC has a total surface area of ~10,800 Å^2^ of which ~7350 Å^2^ or 68% is buried on trimer formation. Such an extensive interface between the three interdigitated polypeptide chains forming this protein explains the high stability of the β-helix to thermal and chemical denaturation described above.

**Figure 5:**
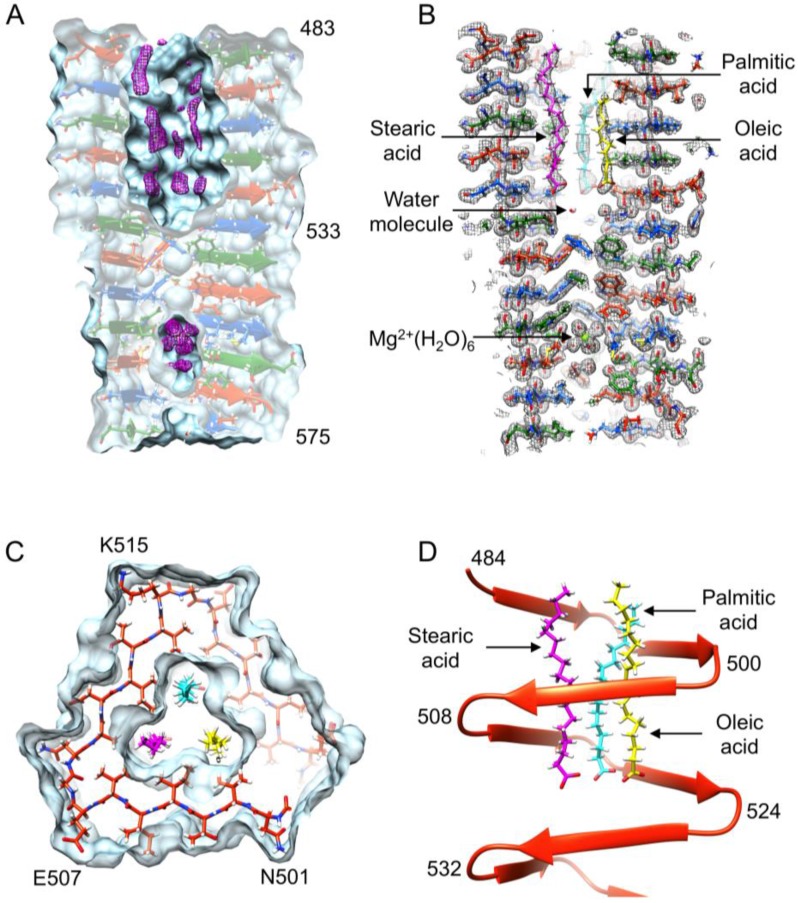
Features of gp5β-BC and gp5β-BC2 structures. (**A**) The Fo-Fc difference map shows that interior cavities are not empty. The difference map (purple) is contoured at 2.5 standard deviations above the mean. The molecular surface is colored aquamarine. Each chain of gp5β-BC is shown in a distinct color (firebrick red, forest green, and dodger blue); (**B**) A 6 Å-thick slice of the refined 2Fo-Fc map is shown with fitted stearic (purple), palmitic (cyan), oleic acids (yellow), and Mg^2+^(H_2_O)_6_ ion. The map is contoured at 1.0 standard deviations above the mean. Carbon atoms are colored in distinct colors, but all oxygens, nitrogens and hydrogens are colored red, blue, and white, respectively; (**C**) Tight packing and interaction of fatty acids with side chains forming the interior of the cavity in gpβ-BC. The color scheme is as in panel B. One complete turn of only one polypeptide chain is shown for clarity. The molecular surface of gpβ-BC is semitransparent light blue; (**D**) Side view of the same chain as in panel (**C**) is shown in ribbon diagram representation.

The N-terminal half of gp5β-BC (residues 483–525) contains a ~18 Å long and ~8 Å wide prism-like hydrophobic cavity that is open to solution at one of its ends. A tube-shaped electron density runs along each of the three edges of the prism through its entire length (Figure 5A). Mass-spectrometry analysis suggested that gp5β-BC contains palmitic, stearic, and oleic acids and their esters (see below). These molecules fit the tube-shaped electron densities well with their head groups at the bottom of the cavity where they form hydrogen bonds with water molecules (Figure 5B). The electron density is of insufficient quality to distinguish them from each other, but one of the electron density “tubes” has a kink roughly in the middle. This tube was assigned to an oleic acid because this molecule is bent due to a double bond roughly in the middle. One of the density tubes is slightly shorter than the two others and it was assigned to a palmitic acid molecule (C16), whereas the longer one was assigned to a stearic acid molecule (C18). The relative ratio or the distribution of the three species in an individual gp5β-BC molecule or in the bulk sample cannot be determined.

The inward-pointing residues of the C-terminal half of gp5β-BC (residues 525–575) are much larger than those of the N-terminal part (Figure 1), and they fill the interior of the β-helix almost completely. Nevertheless, a small hydrophilic cavity, which is completely sealed off from the external medium by the protein atoms, is present roughly in the middle of this hydrophobic stack (Figure 5A). The cavity is occupied by a Mg^2+^ ion and nine water molecules as discussed below (Figure 5B). In the earlier study at 2.9 Å resolution [7], this complex ligand appeared as a single large blob of electron density, which was interpreted as a K^+^ ion because it did not show any anomalous X-ray scattering in the 6–16 keV range.

**Figure 6:**
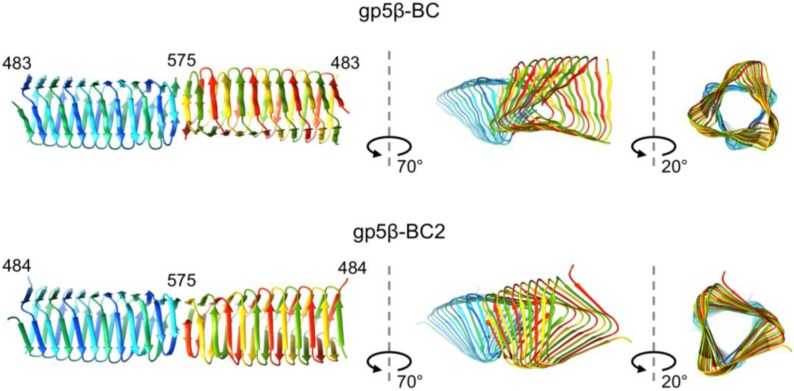
Structure of dimers of trimers of gp5β-BC and gp5β-BC2. Identical interfaces can form markedly different associations of gp5β-BC and gp5β-BC2 into dimers of trimers.

**Figure 7:**
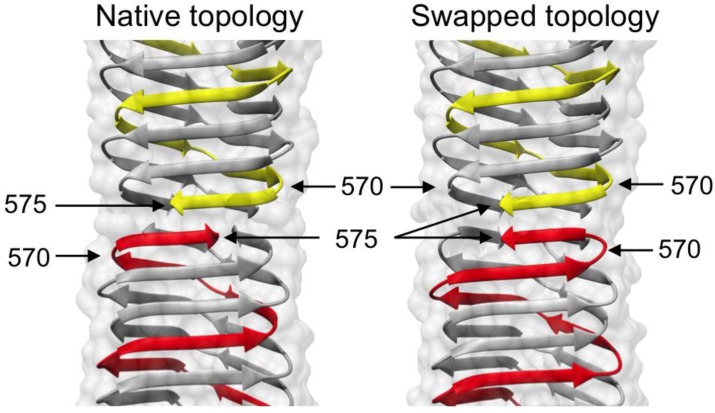
Structural plasticity of the C-terminal tip. The C-terminal β-strand in gp5β-BC2 dimers either retains its native structure (left panel) or undergoes a β-strand swapping (right panel). The wild type structure is found in only one of the three dimers present in the asymmetric unit.

The structure of the gp5β-BC2 trimer is very similar to that of gp5β-BC except for one significant difference at its C terminus. Both, gp5β-BC and gp5β-BC2 trimers interact with each other via their blunt, mostly hydrophobic C-terminal tips forming a dimer of trimers in the crystal. However, the association of gp5β-BC and gp5β-BC2 into dimers differ, despite the interface being formed by the same residues (Figure 6). The two interacting gp5β-BC2 trimers are a smooth extension of each other creating a continuous 24-strand β-sheet, whereas the two gp5β-BC trimers are twisted relative to each other at their interacting interfaces (Figure 6). Interestingly, in one of three gp5β-BC2 dimers present in the asymmetric unit, the last β-strand displays a swapped topology and forms a β-hairpin instead of the native corkscrew-like structure (Figure 7). Remarkably, the corresponding dimer of gp5β-BC2 trimers is virtually identical to the gp5β-BC2 dimer in which both trimers have the native topology. The swapped topology points to possible “breathing” of the C-terminal β-strands before they are locked in the corkscrew configuration by their natural binding partner gp5.4 as shown by the crystal structure of gp5β-BC2/gp5.4 complex (PDB ID 4KU0). Gp5.4 belongs to the class of Proline-Alanine-Alanine-aRginine (PAAR) repeat proteins and forms a membrane-attacking tip of the gp5 spike in the mature phage particle [22].

### 3.5. Identification of the Buried Metal Ion

**Figure 8:**
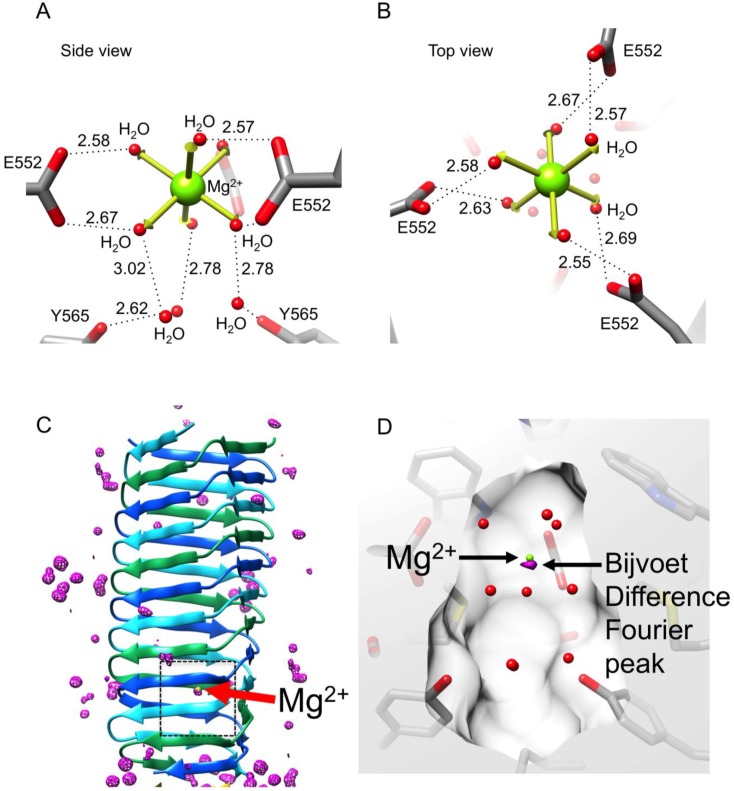
Identification and structure of the buried metal ion. (**A**) and (**B**) Side and top view of the metal ion geometry. The vertices of an ideal octahedron corresponding to waters being 2.16 Å away from the central atom are shown with thick yellow arrows; (**C**) The Bijvoet difference Fourier map (purple) is contoured at five standard deviations above the mean. The black dashed rectangle roughly corresponds to the area shown in (**D**); (**D**) The center of the Bijvoet difference Fourier peak (purple, contoured at 7.5 standard deviations above the mean) does not coincide with the refined position of the Mg^2+^ ion.

A total of nine residues made up of three symmetry-related triplets—E552, S559, and Y565—point toward the hydrophilic cavity in the C-terminal part of gp5β-BC, giving it a net negative charge. The cavity is occupied by a complex structure consisting of a centrally positioned atom surrounded by six ligands 2.16 ± 0.06 Å away and three additional ligands 4.21 ± 0.09 Å away. The six proximal ligands are located at the vertices of an octahedron, in which the root mean square deviation of the bond lengths is 0.25 Å off the perfect octahedron values, Figure 8. These ligands are coordinated by the head groups of three E552 residues. The three distant ligands are coordinated by the O-η atoms of the Y565 residues and form a plane below the octahedron. The geometry of this complex structure suggests that it is a hydrated metal ion of a Me^n+^(H_2_O)_6_ type, which is further decorated with three additional water molecules.

We then tried to identify the metal by analyzing the strength of its anomalous scattering in the Bijvoet Difference Fourier (BDF) synthesis using S atoms of sulfur-containing residues as a reference (three copies of M554 and M557). The anomalous scattering of S at a wavelength of 0.9 Å (the gp5β-BC dataset wavelength, Table 1) is weak at 0.2 electrons but could be detectable in the 1.3 Å resolution gp5β-BC dataset.

The BDF map calculated using the final refined phases contained four non-noise peaks. The highest peak of 8.1σ (8.1 standard deviations from the mean) is centered at the position of the Me^n+^ ion in the Me^n+^(H_2_O)_6_ complex. The other three peaks with heights of 7.1σ, 6.8σ, and 5.5σ corresponded to S atoms in M557 in all the three chains. Of note, sulfurs of other three sulfur-containing residues (three copies of M554) did not produce peaks in this map. Thus, the metal in the Me^n+^(H_2_O)_6_ complex must be a light atom with anomalous scattering comparable or slightly stronger than that of S. Combining this finding with the site’s near perfect octahedral geometry, the only candidate was Ca^2+^, which was present in the crystallization solution as a CaCl_2_ salt. However, none of the crystallographic refinement programs we tried–REFMAC5 [37], PHENIX [41], SHELXL [32]–resulted in a satisfactory refinement of Ca^2+^ in the Ca^2+^(H_2_O)_6_ complex. The difference map always contained a greater than 5σ negative peak suggesting that Ca^2+^ was too electron dense for this position. Besides, the MESPEUS_10 database value for a Ca^2+^–H_2_O bond is 2.46 ± 0.22 Å [42], whereas the site’s metal-water distances were found to be 2.16 ± 0.06 Å (see above). On the other hand, the MESPEUS_10 database value for a Mg^2+^–H_2_O bond is 2.17 ± 0.15 Å. However, anomalous scattering of Mg^2+^ is virtually undetectable at 0.9 Å (0.06 electrons), and Mg^2+^ could not have produced the peak seen in the BDF map. Notably, Mg^2+^ ions were never added to the protein solution at any purification or crystallization step suggesting that this ion should have been present in the cavity from the moment the protein folds.

We also attempted the temperature factor refinement of the metal atom alone while keeping all other parameters fixed. Unfortunately, it turned out to be similarly inconclusive. The Mg^2+^ B-factor of 19.4 Å^2^ was very similar to that of the head groups of the surrounding side chains (20.4 ± 1.4 Å^2^), whereas the Ca^2+^ B-factor of 25.5 Å^2^ resembled that of the surrounding water ligands (25.1 ± 2.9 Å^2^).

A more careful comparison of peak heights in the BDF map with anomalous scattering of Ca and S shows that the peak in the Me^n+^(H_2_O)_6_ complex is too small for a Ca^2+^ ion as its anomalous scattering is 0.48 electrons, which is 2.4 times the strength of S anomalous scattering. The corresponding peak is only 1.3 times greater than the average of S peaks. Hence, the most likely explanation is that over the course of crystallization in a fraction of the molecules a Ca^2+^ ion has diffused into the cavity, which is otherwise completely isolated from the solvent by protein atoms, and expelled the original Mg^2+^ ion. Such an incomplete substitution results in a smaller than expected anomalous signal and contributes little to the appearance of the 2Fo-Fc electron density map.

In order to test this hypothesis, we grew crystals of gp5β-BC in the presence of Sr^2+^ ions and used the much stronger anomalous scattering of Sr atoms at the Sr K-edge energy to determine their location. To aid the analysis of weak Sr sites, these crystals were soaked for several seconds in a cryoprotector solution containing either CaCl_2_ or SrCl_2_ in addition to the other crystallization solution compounds (MPD and Na-Acetate) prior to data collection. For both types of crystals (Sr-grown/Sr-cryo and Sr-grown/Ca-cryo), the BDF map showed an above the noise level peak near the central atom position, but not overlapping with it (Figure 8C,D). In both datasets, the BDF peak was shifted from the 2Fo-Fc map peak by 0.49 ± 0.01 Å along the threefold axis towards the geometrical center of the cavity (Figure 8D). Notably, the 2Fo-Fc electron density maps of the Sr-grown crystals were essentially identical in terms of the site’s geometry and bond distances to that of the original Ca-grown crystal and they showed an excellent density for all the water ligands surrounding the central metal atom site.

These findings are in agreement with the previously proposed hypothesis. During crystallization, Sr^2+^ ions from the crystallization solution were indeed able to diffuse into the cavity thus expelling the original Mg^2+^ ions. However, this happened only in a small fraction of protein molecules. If we use the height of a BDF peak as a site occupancy estimate, Sr substitutes Mg in only about 7% of the molecules, whereas the rest of the molecules still contain the original Mg^2+^(H_2_O)_6_ ion. As a result, the contribution of this Sr atom to the 2Fo-Fc synthesis is negligibly small, and the 2Fo-Fc electron density map of the Sr-grown crystals shows a Mg^2+^(H_2_O)_6_ ion. Furthermore, the cavity is large enough to accommodate a Sr^2+^(H_2_O)_6_ ion. However, as the Sr^2+^–H_2_O bond is longer than the Mg^2+^–H_2_O bond, the Sr^2+^ ion binds slightly further away from the head groups of the E552 residues and the BDF map shows its exact location. In summary, similar to the original Ca-grown crystals, the anomalous scattering signal of a low occupancy Sr atom is easily detectable in a BDF map, but is lost in the 2Fo-Fc electron density map, which shows the predominant species–a Mg^2+^(H_2_O)_6_ ion. Also of note is that crystallized protein appears to be able to “breathe” and can exchange buried ions for ions in the surrounding solution.

### 3.6. Identification of the Internal Compounds

The hydrophobic nature of the gp5β-BC N-terminal cavity suggested that the molecules buried in it—the internal compounds—must be also mostly hydrophobic. The tubular appearance and volume of the corresponding electron densities implied that these molecules have a linear structure and that their mass is unlikely to exceed 300 Da. Furthermore, the electron density of the inclusion compounds was significantly weaker than that of the polypeptide chain suggesting either incomplete occupancy, crystal averaging of heterogeneous compounds, or disorder. Because of the size and non-covalent interaction of these molecules with the protein, we attempted to identify them with the help of ElectroSpray Ionization Mass Spectrometry (ESI-MS) employing different ionization conditions and sample preparation methods. Nondenaturing ESI-MS is an established technique for determination of protein–ligand interactions, such as detecting ligands in orphan nuclear receptors [43] or pharmaceuticals in proteins [44], and for characterization of noncovalent complexes [45,46,47].

**Figure 9:**
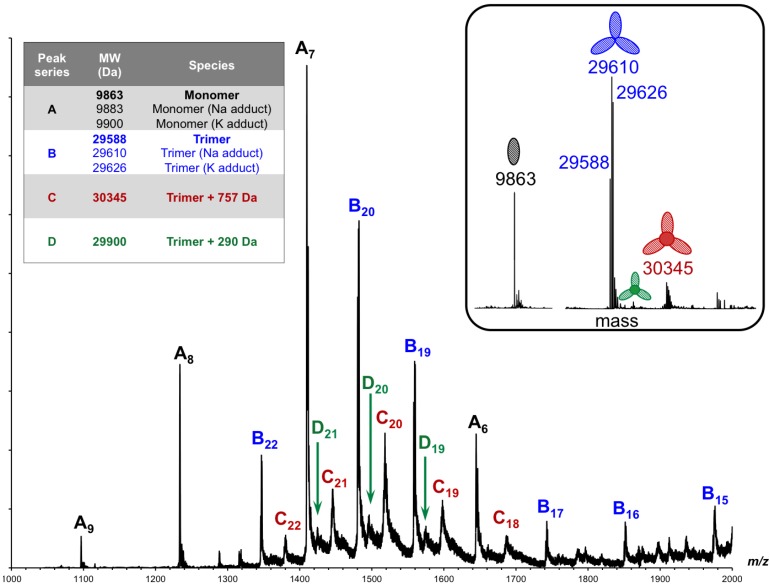
ESI-QTOF mass spectra of gp5β-BC acquired in smooth ionization conditions. The sample cone voltage and the collision energy were 35 V and 4 eV, respectively. The inset shows the deconvoluted mass spectrum. The peak A series corresponds to the mass of the monomer with H, Na and K adducts, the peak B series to the mass of the “empty” trimer, and peaks C and D series to the mass of the trimer with inclusion compounds.

Initially, we analyzed gp5β-BC by ESI-MS under soft ionization conditions in the so-called “smooth” ESI mode (low sample cone voltage and collision energy). The sample was found to contain several species (Figure 9): a monomer (family of peaks A, 9863 Da), an empty trimer (family of peaks B, 29,588 Da), a trimer with an additional mass of 757 Da (family of peaks C, 30,345 Da), and a trimer with additional 290 Da (family of peaks D, 29,900 Da).

**Figure 10:**
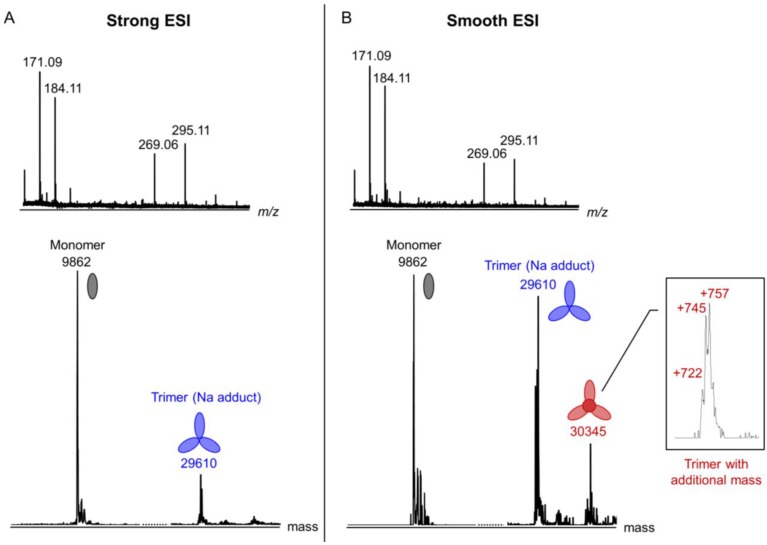
ESI-QTOF analysis of gp5β-BC under different ionization conditions. Strong and smooth ionization conditions are shown in (**A**) and (**B**), respectively. The inset shows the peak corresponding to a protein species with additional mass (putative inclusion compounds) in greater detail. The ionization conditions are as follows: the sample cone voltage is 100 V and the collision energy is 15 eV for Strong ESI; the sample cone voltage is 35 V and the collision energy is 4 eV for Smooth ESI.

We then changed the experimental conditions to facilitate dissociation of the trimer and release of internal compounds. The sample cone voltage and collision energy were increased (the “strong” ESI mode), the sample was heated to 95 °C or diluted into a denaturing buffer (1% formic acid in 1:1 water/acetonitrile). All experiments had similar results demonstrating a decrease in the amount of the empty trimer and an increase in the amount of the monomer (Figure 10). However, no significant difference in the low mass range of the spectra can be seen either in the positive ionization mode (Figure 10A) or in the negative mode (not shown). This suggests that the internal compounds are not ionized in either of the ionization modes or that only the empty trimer dissociated into monomers, but not the trimer containing the internal compounds. Depending on the protein batch and ionization conditions, the mass of the trimer with the internal compounds varied from 30,310–30,333 and 30,345 Da, which corresponds to the mass increase of 722,745 and 757 Da, respectively. It is therefore possible that several different molecules can be accommodated inside the trimer.

**Figure 11:**
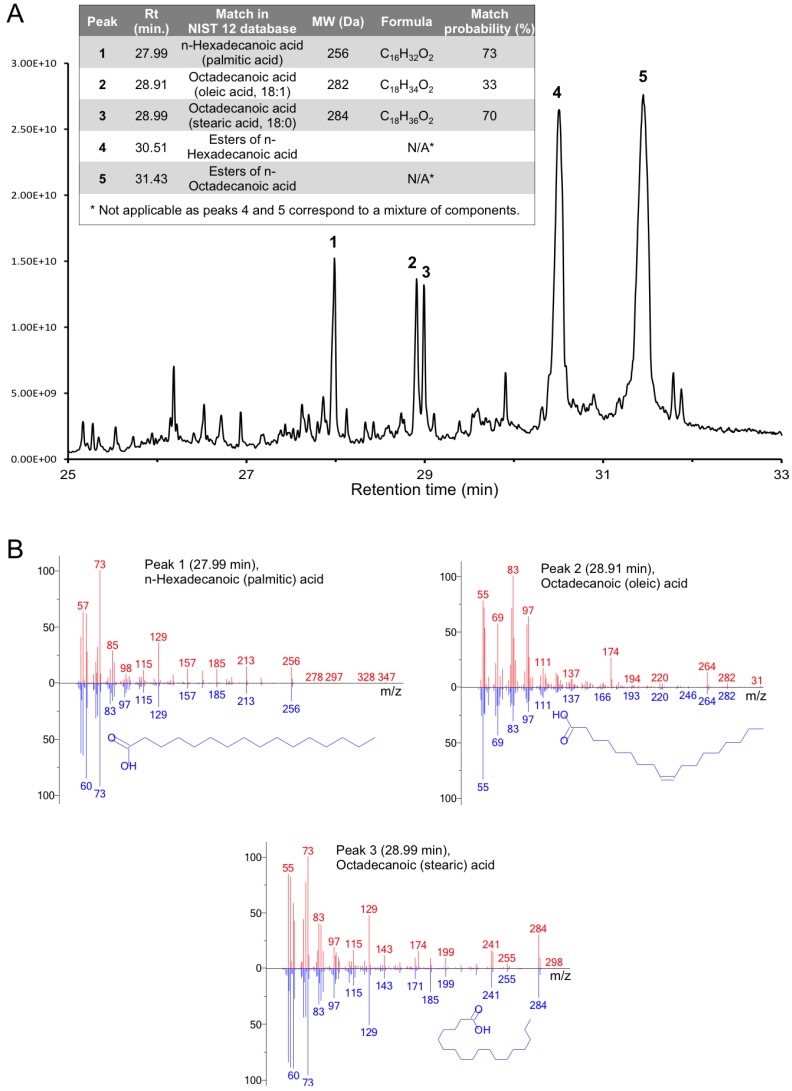
Identification of the inclusion compounds by GC-MS chromatography of gp5β-BC organic extract. (**A**) GC-MS chromatography of gp5β-BC organic extract; (**B**) Comparison of the Electron Impact (EI) spectra of the GC-MS compounds with NIST 12 database. The EI mass spectra of Peaks 1, 2 and 3 eluted respectively at 27.99, 28.91 and 28.99 min match those of the palmitic acid, oleic acid, and stearic acid, respectively.

The low mass peaks that characterize the inclusion compounds are very broad indicating heterogeneity (Figure 10B, insert). We attempted to reduce the low mass contaminants by purifying the protein by crystallization. Crystals were collected, washed in the protein-free crystallization solution, and dialyzed against pure water (see Materials and Methods). Unfortunately, this procedure did not lead to a significant improvement in the quality of the low mass part of the spectrum, and the peaks remained broad. Nevertheless, this “*in crystallo*” purified sample was then used for chloroform/methanol organic compound extraction followed by gas chromatography-mass spectrometry (GC-MS) analysis (see Materials and Methods). A similar procedure was performed by Potier *et al.* 2003 [43]. The GC-MS chromatogram had five significant peaks, which were matched against the National Institute of Standards and Technology Database (Data version: NIST 14) [48] (Figure 11). Three fatty acids could be identified in the organic extract with a high confidence: 16:0 n-hexadecanoic acid (palmitic acid), 18:0 octadecanoic acid (stearic acid) and 18:1 octadecenoic acid (oleic acid). Two other major could correspond to esters of hexa- and octadecanoic acids, but the probability scores are low and could not allow for a better identification. Most likely, these peaks contain two or more co-eluted compounds. This heterogeneity is consistent with broad peaks seen in other MS experiments.

In summary, the MS analysis did result in a precise identification of internal compounds but showed that these compounds are a mixture of fatty acids and/or their derivatives (such as esters). As a final step in the internal compound identification, the atomic models of the stearic, oleic and palmitic acids were placed into the corresponding electron densities inside the hydrophobic cavity of gp5β-BC and refined. Taking into account that the electron density of the internal compounds was weaker than that of the protein, the refinement can be considered as successful. No peak above 3.5 RMSD in the Fo−Fc difference map is present, the geometry is good, and the density fit is satisfactory (Figure 5B).

### 3.7. Full-Length Gp5 Contains Fatty Acids

The presence of fatty acids in the gp5β-BC structure made us reexamine the structure of full-length gp5, which was solved to 2.9 Å resolution earlier [7]. Similar tubes of electron density were present in the cavity of full-length gp5, albeit these densities were even weaker than those in the gp5β-BC structure. Of note, the electron density of fatty acids was virtually undetectable in a 3.4 Å resolution dataset of gp5β-BC2 with a bound PAAR-repeat protein (PDB ID 4JIW) [22] but was much better defined in a 1.15 Å resolution dataset of the gp5β-BC2/gp5.4 complex (PDB ID 4KU0), showing that high resolution terms are important for revealing these ligands.

**Figure 12:**
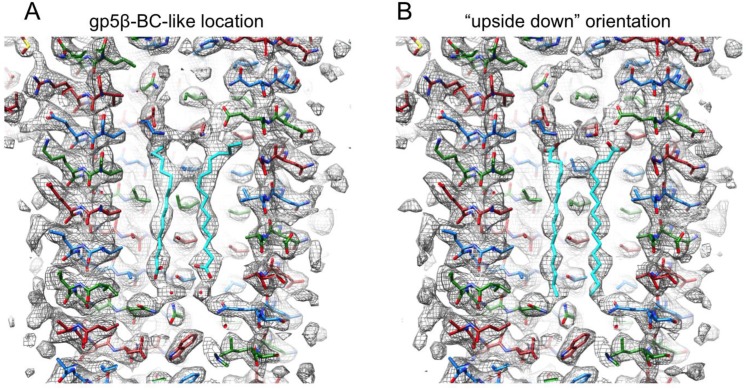
Fit of the palmitic acid molecule into the electron density of the full-length gp5 protein (PDB ID 1K28). (**A**) The location and orientation of the palmitic acid molecule are similar to those in the gp5β-BC structure; (**B**) The palmitic acid molecule is in an “upside down” orientation. Both 2Fo-Fc maps are contoured at 1.0 standard deviations above the mean. The color scheme is as in Figure 5B.

Despite the low quality of electron density, the shortest of the gp5β-BC fatty acids—a palmitic acid—matched the electron density reasonably well. We did not attempt to model other acids because the asymmetric unit in the full-length gp5 structure contains only one gp5 polypeptide chain and thus only one independent fatty acid. The poor quality of electron density made it difficult to distinguish the orientation of the palmitic acid molecule, as well as its exact location along the electron density tube. We performed several rounds of refinement with the palmitic acid in different starting positions and orientations. The crystallographic refinement parameters were similar to those obtained in trials with an empty cavity or cavity filled with water molecules. Two best (in terms of density fit analysis) refinement trials are shown in Figure 12. In one case, the position and orientation of the palmitic acid are similar to those found in the gp5β-BC structure. In the other case, the molecule is in an “upside down” orientation. Density fit analysis strongly favors the original orientation.

## 4. Discussion

More than 78% of the complete gp5 β-helical domain—18 out of 23 β-strands (Figure 1)—is fully interdigitated with each amino acid in a given polypeptide chain interacting with two amino acids from the same chain (within each β-strand) and four to six amino acids from two *other* polypeptide chains forming the trimer (the β-strands immediately preceding and succeeding the current β-strand). Clearly, the folding and formation of secondary structure in a gp5-like β-helix must depend on the correct, precisely timed, and in-register association of the three chains. This means that folding of an individual chain of a gp5-like β-helix cannot take place separately from trimer formation. This situation is very much unlike other oligomeric proteins, including interdigitated all-α-helical proteins, where the secondary and in most cases tertiary structure is formed first, which is then followed by the formation of the quaternary structure. In gp5 β-helix, the secondary, tertiary, and quaternary structures are inseparable. Therefore, many established protein folding principles and concepts must be applied to gp5 β-helix with caution.

Biophysical and structural characterization of the gp5 β-helix presented here makes it possible to identify the region that likely initiates trimerization and thus folding of this protein with an unusual intertwined topology. Because the gp5β-B fragment (residues 483–525, Figure 1) has a defined oligomeric state and β-structure in solution, but the gp5β-C fragment (residues 523–575, Figure 1) is not β-structural, the sequence sufficient for folding initiation is contained within residues 483–525 (Figure 2).

A C-terminal part is critical for folding of most fibrous beta-structural and collagenous, as well as many coiled coil proteins [49]. There are many examples of soluble, correctly folded C-terminal domains of fibrous proteins [12,50,51,52,53], but very few instances of oligomeric, properly folded middle or N-terminal domains [52,54]. Furthermore, many fibers carry a folding chaperone that either forms a C-terminal domain, which is cleaved off in the mature protein, or encoded by a separate gene immediately downstream from the fiber gene [55,56]. In all of these cases, the C-terminal domain is likely important for creating an in-register arrangement of the three partially folded chains, which then complete their folding upon association into a trimer.

Full-length gp5 follows this principle and its C-terminal domain is responsible for trimerization of the entire protein. However, the β-helix alone, if considered as a fibrous protein, does not obey this rule, mostly likely because of its highly interdigitated topology. Its C-terminal tip part (the gp5β-C fragment) is unable to fold independently despite having a very well packed hydrophobic interior, buried glutamates, and a metal ion that could function to keep the three chains in-register during folding. Instead, the “folding nucleus” of gp5 β-helix is shifted slightly to the N terminus (residues 483–525) because the gp5β-B fragment can fold. This portion could be considered as an autonomous folding unit, which is termed “trigger sequence” in coiled coil proteins [57]. This part of the protein thus appears to be responsible for trimerization of gp5 β-helix and of the whole gp5 protein. Properly folded gp5 is needed for assembly of the baseplate hub, which is in turn required for assembly of T4 tail [21]. Hence, folding and trimerization of residues 483–525 of gp5 determine the morphogenesis of the phage T4 particle. This region has the strongest sequence repeat compared to the rest of the β-helix. This feature could be an important factor that determines its self-assembly properties because the strong repeat manifests itself in a very regular structure (Figure 1). Residues in the second position of the repeat—either N or D—form a continuous hydrogen-bonded ladder on the surface of the β-helix. Residues in the last repeat position—either E or K—alternate and create salt bridges that also span the entire structure. Interestingly, the “folding nucleus” of gp5 comprises the walls of the fatty acid-binding cavity, but does not include its bottom. Assuming gp5β-B fragment has a structure similar to that found in the larger fragment it might be capable of binding fatty acids or similar ligands that could contribute to its association into a dimer of trimers.

Our data indicate that fatty acids are not required for maintaining the structure of gp5 β-helix. Indeed, guanidinium at low concentrations does not disturb the structure of gp5β-BC (Figure 3C and Figure 4B) but it profoundly decreases the intrinsic fluorescence of the protein (Figure 4A). This could be explained by the fatty acids leaving their cavity and exposing it to solution with the protein preserving its structure. Most of gp5 β-helix fluorescence is due to the nine stacked tryptophans (three copies of W536, W544 and W550) that form the bottom of the fatty acid binding cavity. Initially, the fatty acids shield the tryptophans from the solvent (water molecules near the head groups of the fatty acids do not contribute to fluorescence quenching, as they are immobile). However, even at low concentrations, guanidinium is likely to cause the fatty acids to leave the cavity thus opening it to solvent and leading to fluorescence quenching.

**Figure 13:**
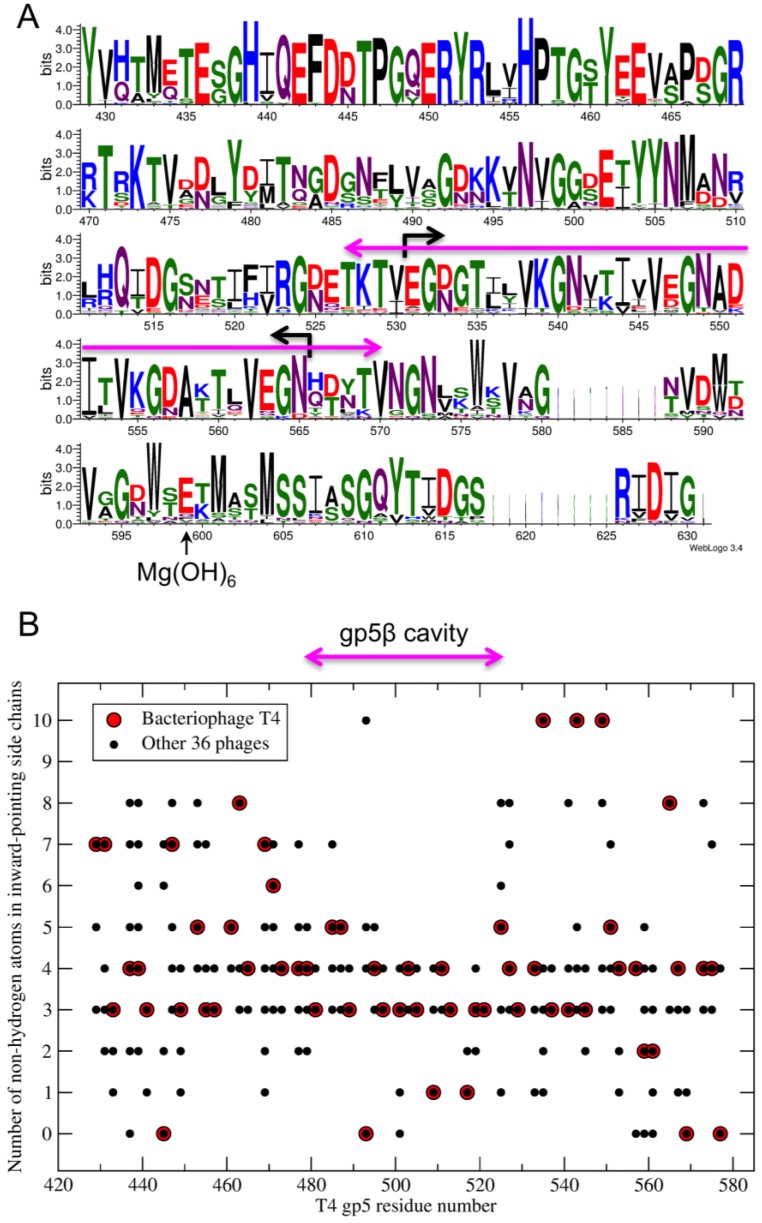
Sequence conservation analysis of gp5 β-helix. (**A**) Sequence conservation of gp5-like β-helices is shown with the help of the WebLogo program [58] in which the height of the letter is proportional to the degree of conservation in this position. The purple arrow corresponds to the cavity in gp5. The black arrows show the borders of the gp5β-B fragment; (**B**) The size of inward-pointing side chains in gp5-like β-helices is evaluated as a number of non-hydrogen atoms. The same alignment, which was calculated with the help of ClustalW program [59], was used in both panels. Gene IDs are given in Materials and Methods.

Our data also demonstrate that heat-induced unfolding of the β-helix in the presence of guanidinium is irreversible. This suggests that fatty acids (and possibly the buried Mg^2+^ ion) could be required for folding. Guanidinium is likely to inhibit the interactions of the protein chains with fatty acids and/or the Mg^2+^ ion, thus preventing refolding as the temperature is lowered (Figure 3C). Unfortunately, we were unable to find conditions to test the function of fatty acids and Mg^2+^ ions in folding of the β-helix. Such a refolding experiment requires removal of guanidinium with a simultaneous addition of fatty acids and/or Mg^2+^ ions.

Alignment of gp5-like β-helices shows that the overall conservation decreases toward the C terminus (Figure 13A). Nevertheless but unsurprisingly, large hydrophobic residues pointing inwards (tryptophans and methionines), the VxG repeat, and the last β-strand, which is responsible for binding gp5.4, all show a high degree of conservation. Interestingly and despite the low overall sequence identity, all proteins appear to contain a cavity that is similar in terms of size, location in the protein, and properties of amino acids forming its walls, to that found in T4 gp5 (Figure 13B). It is therefore possible that all these proteins carry fatty acids inside their gp5 β-helices or fatty acids participate in folding of these proteins.

**Figure 14:**
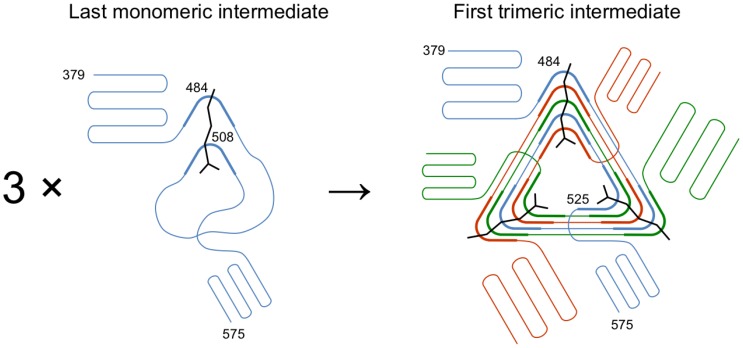
Trimerization of gp5 β-helix. Binding of a fatty acid (black) to a polypeptide chain (blue) promotes formation of a characteristic kink region (thicker line) and a corkscrew-like topology in a single chain. In the next step, fatty acids participate in the association of three chains into a trimer by facilitating the formation of kinks in other chains. Regions with a zigzag-like β-sheet topology are formed independently of the presence of fatty acids. Residue numbers are given for strategic locations.

The antiparallel β-sheet topology of the N-terminal part of the complete gp5 β-helical domain (Figure 1) and of the swapped C-terminal β-strand in the gp5β-BC2 trimer (Figure 7) suggests that these parts of the polypeptide chain have an intrinsic propensity to form a single-chain antiparallel β-sheet, which could constitute their first folding intermediate. Presumably, a newly synthesized gp5 polypeptide forms these partially folded segments prior to the trimerization of its folding nucleus or “trigger sequence”, which stays completely unfolded at this initial stage. The question of how the trigger sequence folds and trimerizes at the same time is more difficult to answer. A monomer with a close-to-native corkscrew-like structure is unlikely to form in solution because it completely lacks long distance interactions (Figure 5D). Such an intermediate, however, can be stabilized by a fatty acid or another extended molecule of appropriate length, which will provide the needed long distance contacts (Figure 14). This moiety can bind to the kinked regions that could form spontaneously because they are created by intra-chain interactions (Figure 5C). When three chains are in close proximity, the fatty acid will promote the formation of the kinks in the neighboring chains while simultaneously coiling and interdigitating the three β-strands. Thus, fatty acids could play an important role in registering the three chains and nucleating the folding of the gp5 β-helix (Figure 14).

The triple-stranded β-helix of gp5 is one of very few known soluble oligomeric fibrous proteins that have a well-defined size, low propensity to aggregation, and high resistance to harsh chemical and physical treatment [51,60,61,62]. These properties make gp5 β-helix a useful bioengineering tool. It has already been used as a trimerization motif [22] and as a chemical reaction template [25,63]. The experiments reported here suggest that future applications should focus on the gp5β-B fragment (residues 483–525) because it is soluble, can self-assemble into a very stable structure, and has six termini that are open to solution and can be functionalized. Furthermore, this fragment shows a strong repeat making it possible to change its length and thus fine-tune the desired architecture.

## 5. Conclusions

The biophysical and biochemical properties of a fully interdigitated, trimeric, β-helical protein described here are as unusual as is its structure. Buried fatty acids can be detected in mass-spectrometry analysis and can explain the pronounced increase in fluorescence during denaturation. However, their density in crystallographic analysis is very weak and their location in the electron density map is thus ambiguous. The buried metal ion is fully shielded from solvent but nevertheless can be kicked out from its cavity by an ion from the crystallization solution. The role of either the fatty acids or the metal ion in phage T4 morphogenesis or infection is also unclear. In summary, the gp5 β-helix is a small but fascinating protein that has evolved to perform the unique function of a membrane drill bit, which might explain its unusual properties.
